# Silicone soft socket system for the treatment of geriatric transtibial amputees

**DOI:** 10.1007/s00508-020-01643-8

**Published:** 2020-04-16

**Authors:** Robert Breuer, Klemens Trieb

**Affiliations:** 1 Department of Orthopedics and Trauma Surgery, General Hospital of Vienna, Waehringer Guertel 18–20, 1090 Vienna, Austria; 2 Computed Tomography Research Group, University of Applied Sciences Upper Austria, Stelzhamerstraße 23, 4600 Wels, Austria

**Keywords:** Outcome, Silicone liner, Walking distance, Duration of daily use, Satisfaction

## Abstract

**Background:**

Outfitting geriatric amputees with a suitable prosthesis is a demanding task. The aim of this study was to determine the effect of prostheses outfitted with a silicone suspension interface system on a large group of transtibial amputees regarding walking distance, satisfaction of use and duration of daily use.

**Methods:**

The study included 75 mostly geriatric transtibial amputees fitted with a rigid total contact socket with a silicone interface system called an Icelandic roll-on silicone socket (ICEROSS, Ossur, Reykjavik, Iceland). At follow-up 54 patients remained for assessment. Ambulatory performance was measured by first categorizing the patients into four subgroups regarding their walking capacity: 0–50 m, 50–100 m, 100–500 m and >500 m. The alteration in patient mobility was measured at admission, discharge and follow-up. Satisfaction and duration of daily use as well as use of walking aids were collated with a standard questionnaire.

**Results:**

Between admission and discharge, patients showed significant improvement (*p* = <0.001) in walking distances. The walking distance diminished again between time of discharge and follow-up (*p* = <0.001). The parameters satisfaction with device and duration of daily use showed no significant differences and the same applied to male and female subjects.

**Conclusions:**

Geriatric transtibial amputees fitted with prostheses attached via a silicone suction socket system showed significant improvements in walking distances and a high rate of satisfaction with the device. There were no statistically significant gender-specific differences among users of the ICEROSS system.

## Introduction

The loss of a lower limb due to amputation is often the end of a long period of illness for older patients [3, 6]. Therefore, it poses a physically as well as psychologically demanding situation [6, 18]. Rehabilitation of these patients is a challenge, but nevertheless indispensable in order to enable the amputees to participate in everyday life. A major part of this rehabilitation process is the provision of a fitting and easy to use prosthesis system [12]. When designing and developing a suitable prosthesis many different problems, such as pistoning, extensive rotation and translation, walking pattern, qualities of residual skin and soft tissue have to be taken into account [10, 11]. A major role in addressing these issues depends on selecting an appropriate suspension system [8, 9]. In the late 1980s, the USA and Iceland developed new models of silicone suspension systems, including the 3‑S system [7] and the Icelandic roll-on silicone socket (ICEROSS system, IRS [ICEROSS, Ossur, Reykjavik, Iceland]) [14] to address these problems. The effects of these systems have already been proven for transfemoral amputees [16] and even transtibial amputees [5]. Nevertheless, systematic reviews do not support liner systems as a general solution without reservation [4, 13]. This study of 75 transtibial amputees aimed to show the effect of prostheses fitted with a silicone suspension interface (ICEROSS, Ossur, Reykjavik, Iceland) on walking distance, satisfaction of use and duration of daily use in a large patient group.

## Patients, material and methods

### Material and methods

In this retrospective study the walking distances of 75 mostly geriatric transtibial amputees treated at an orthopedic rehabilitation center, were analyzed. Written informed consent was obtained from all participants. The patients were amputated in a hospital 4–8 weeks before admission to the rehabilitation center after wound healing and proper stump management were ensured. The reason for amputation was always chronic vascular problems and subsequent development of dry or wet gangrene. No subjects after amputation due to traumatic reasons were included. All patients were novel prosthesis users and provided primarily with prostheses with a rigid total contact socket augmented by a silicone interface system (IRS). The mean age of the subjects was 68 years (±9.6 years; range 43–87 years). Duration of stay at the rehab averaged 34 days (±8.9 days). The group consisted of 43 women and 32 men. Walking distance was estimated over a standard course commonly used in the rehabilitation center. To specify the walking distance, the patients’ performances were categorized into four groups based on distance traversed: 0–50 m, 50–100 m, 100–500 m, and >500 m. The change in walking distance was measured at admission, discharge and follow-up. The follow-up was 8.5 (±3.1) months after dismissal from the rehab stay, 54 patients could be included and 2 patient had died (Table 1). The rehab center was well known all over the country for rehabilitation after amputation. Most of the patients were older persons and often had limited means of contact opportunity, therefore,19 subjects could not be reached, which were lost to follow-up due to a lack of valid contact data. The measurement of satisfaction and time of daily use were collated with a standard questionnaire routinely used in the rehab center. It consisted of three gradations (satisfied, moderately satisfied, not satisfied), from which the patient should pick the one option, which depicted their subjective content with the supplied prostheses. Each amputee was examined clinically by an orthopedic surgeon of the rehab institution.

**Table 1:** Descriptive data of participating subjects

Number (*n*)	75
Follow-up (*n*)	54
Age (years)	68 ± 9.8
Women/men	43/32
Duration of rehab (days)	34.17 ± 8.9
Follow-up time (months)	8.6 ± 3.0
Repair	22%

### Statistics

Group differences between male and female patients with respect to the walking distance were tested for significance by the nonparametric Mann-Whitney U‑test. Differences between time of admission, discharge and follow-up regarding the walking distance were statistically analyzed by the nonparametric Friedman test. The cut-off level for statistical significance was set at *p* < 0.05, 2‑tailed. For statistical comparisons, the distance categories were given the values 1–4. The difference between the assigned values were calculated for each participant and used as a measure for change in mobility between the points of follow-up. All statistical analyses were carried out by SPSS for Windows, version 11.0 (SPSS Inc., Chicago, IL, USA).

## Results

### Walking distance and walking aids

To properly assess the ambulation performance, the patients were subdivided into four groups, regarding their maximal walking distance: 0–50 m, 50–100 m, 100 to 500 m, and >500 m. A significant improvement in mobility was definitely achieved by prosthetic provision during rehabilitation as can be observed by comparing the total numbers of patients advancing into superior groups (*p* = <0.001) (Table 2).

**Table 2:** Number of patients and their respective walking distances at admission, discharge and follow-up

Walking distance (m)	0–50	50–100	100–500	>500
Admission	10	56	7	2
Discharge	4	23	23	25
Follow-up	20	0	32	2

**Fig. 1:**
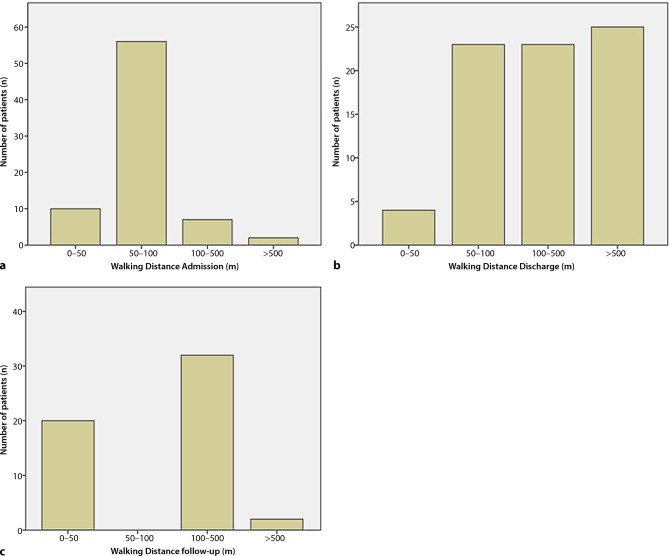
**a** Number of patients sorted by walking distance at admission, **b** number of patients sorted by walking distance at discharge, **c** number of patients sorted by walking distance at follow-up

At admission more than 88% (66/75) of the subjects could not walk further than 100 m (Fig. 1a). This group could be reduced to 36% (27/75) at time of discharge (Fig. 1b).

Interestingly, when comparing the number of patients in the group with walking distance >500 m between time of discharge and follow-up, the percentage decreased from 33% (25/75) to 4% (2/54, *p* = <0.001). At follow-up, three patients did not use their prosthesis at all. In conclusion, significant differences with respect to the walking distance were found between the three times of evaluation (χ^2^ = 31.4; d = 2; *p* ≤ 0.0001). The shortest distances were observed at admission, and the longest distances at time of discharge. Furthermore, a statistical trend between males and females with respect to the walking distance could be found at time of follow-up (z = −1.759; *p* = 0.078). Males were more likely to walk longer distances than females. No significant differences in ambulatory performance between male and female patients were found at the times of admission (z = −0.710; *p* = 0.478) or discharge (z = −1.321; *p* = 0.187).

### Time of use and satisfaction

**Fig. 2:**
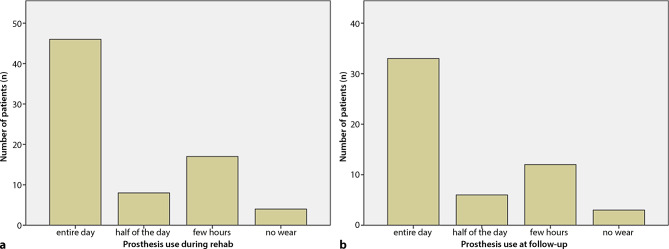
**a** Number of patients sorted by duration of prosthesis use during rehab,** b** number of patients sorted by duration of prosthesis use at follow-up

**Fig. 3:**
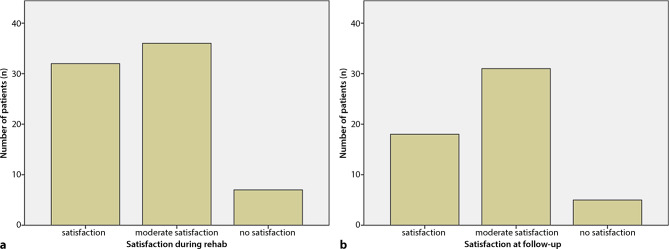
**a** Number of patients sorted by satisfaction with the supplied prosthesis at rehab,** b** number of patients sorted by satisfaction with the supplied prosthesis at follow-up

While at rehab, the prosthesis was used by 62% (46/75) of the patients during the entire day, and 11% (8/75) for half of the day, 25% of the subjects (17/75) wore the device only for a few hours; 5% (4/75) completely neglected the prosthesis. At follow-up, 61% of the subjects (33/54) used the prosthesis the entire day, 11% (6/54) half of the day, 22% (12/54) only for a few hours and 6% (3/54) not at all. A share of 22% of the prostheses required some kind of repair within the observation period. Regarding acceptability of the supplied prostheses during rehab, 33% (25/75) of the amputees reported satisfaction, 57% (43/75) moderate satisfaction, and 9% (7/75) no satisfaction. At time of follow-up the numbers remained the same with 33% (18/54) satisfied, 57% (31/54) moderately satisfied, and 9% (5/54) not satisfied (Figs. 2 and 3).

The use of walking aids was reduced remarkably from the time of admission to the time of discharge as shown in Table 3. Especially the use of wheelchairs could be reduced significantly and an overall downgrade to lower levels of walking aids could be observed.

**Table 3:** Walking aids used at time of admission and time of discharge

Walking aids	Wheelchair	Walking frame	2 crutches	1 crutch
Admission	10	10	43	6
Discharge	0	13	31	11

## Discussion

Providing elderly or even geriatric patients with prostheses results in certain challenges that need to be addressed and resolved. Atrophic skin conditions with the possibility of stump skin breakdown, reduced capacity of wound healing because of multimorbidity and vascular or metabolic disease are only an excerpt of a various number of issues which have to be taken care of [8, 16]. In our study we present the results of a large cohort of transtibial amputees provided with a prosthesis outfitted with a silicon liner system (IRS).

To resolve some of these issues the silicone liner suspension systems were developed in the USA and Iceland in the late 1980s [7, 14]. These systems have a lot of advantages, such as good adaptation and adhesion onto the stump, an even force distribution to the prosthesis socket and enhanced comfort in comparison to other systems [2, 5]. Within our analysis of transtibial amputees, we were able to illustrate considerable improvement concerning ambulating distance during rehabilitation and provision with an IRS system at a low rate of necessary repairs compared to other interface systems used in the past. The dependence on walking aids and especially wheelchairs was significantly diminished, and the duration of daily use as well as the satisfaction with the prosthesis increased. One of the big advantages, which probably contributes to all of these findings, is the possibility of attaching the prosthesis while the patient is in a sitting position. This might seem to be a rather unimportant detail, but greatly facilitates the process of attachment for geriatric patients. It has to be taken into account that putting on a prosthesis while standing requires a certain skill and balance, therefore the sitting position with the IRS system offers a safe option for elderly people. The latest improvement of the IRS system consists of a hook and loop fastener (HOLO system), which not only facilitates donning and doffing even further, but also improves mechanical properties during fast gait [1].

Interestingly, at follow-up the group with distance ambulated >500 m, which was built up during rehabilitation, decreased. Yet patients reported similar rates of acceptance (33% satisfied and 57% moderately satisfied) and also a long time of daily use (entire day 61%). That leads to the assumption that the perceived reduction of walking-distance may be explained by reduced therapy and walking training. Nevertheless, systematic reviews exhibited a certain scepticism concerning the positive effects of silicone liner systems for transtibial amputees [4, 13].

The strengths of this study are the relatively high number of included subjects and homogeneous patient group, which consisted of mostly geriatric, multimorbid patients who were amputated solely because of non-traumatic reasons and were novel prosthetic users.

Limitations of our study are of course the retrospective study design and the lack of a control group. Moreover, the effects of physiotherapy and prosthetic outfitting were not defined separately.

In our study we were able to show a very good rehabilitation result regarding walking distance, comfort of wear and time of use. These facts support the overall tendency that the use of silicone liners, especially the IRS system, have been a milestone in prosthetic technological development and happened to be a well-established piece of prosthetic outfitting. Silicone liners as a gold standard of prosthetic to limb interface, are still under development. For example, existing problems with heat dissipation and consecutive sweating and compromised skin are topics of current research [17] as well as further improvement of prosthetic fitting [15].

Further studies have to be conducted, prospective and randomized and especially with control groups, to evaluate the definite role of silicone liner systems such as IRS for transtibial amputees.

## Conclusion

Supplying elderly patients with IRS provides considerable benefits. The higher costs of purchase compared to other liner systems are compensated by a high satisfaction and comfort of wear which reduced subsequent expenditure because of lower frequencies of necessary adjustments, the use of additional walking aids and a high acceptability of the prostheses.

## References

[1] Abu Osman NA, Gholizadeh H, Eshraghi A, Wan Abas WAB (2017). Clinical evaluation of a prosthetic suspension system: looped silicone liner. Prosthet Orthot Int.

[2] Ali S, Abu Osman NA, Naqshbandi MM, Eshraghi A, Kamyab M, Gholizadeh H (2012). Qualitative study of prosthetic suspension systems on transtibial amputees’ satisfaction and perceived problems with their prosthetic devices. Arch Phys Med Rehabil.

[3] Amtmann D, Morgan SJ, Kim J, Hafner BJ (2015). Health-related profiles of people with lower limb loss. Arch Phys Med Rehabil.

[4] Baars ECT, Geertzen JHB (2005). Possible advantages of silicon liner socket use in trans-tibial prostheses. Prosthet Orthot Int.

[5] Dasgupta AK, McCluskie PJ, Patel VS, Robins L (1997). The performance of the ICEROSS prostheses amongst transtibial amputees with a special reference to the workplace—a preliminary study. Occup Med (Lond).

[6] Dillingham TR, Liliana EP, MacKenzie EJ (2002). Limb amputation and limb deficiency: epidemiology and recent trends in the United States. South Med J.

[7] Fillauer C, Pritham C, Fillauer K (1989). Evolution and development of the silicone suction socket (3S) for below-knee prostheses. J Prosthet Orthot.

[8] Gholizadeh H, Abu Osman NA, Eshraghi A, Ali S, Razak NA (2014). Transtibial prosthesis suspension systems: systematic review of literature. Clin Biomech.

[9] Gholizadeh H, Abu Osman NA, Lúvíksdóttir ÁG, Eshraghi A, Kamyab M, Wan Abas WAB (2011). A new approach for the pistoning measurement in transtibial prosthesis. Prosthet Orthot Int.

[10] Gholizadeh H, Osman NAA, Kamyab M, Eshraghi A, Abas WABW, Azam MN (2012). Transtibial prosthetic socket pistoning : static evaluation of seal-in ® X5 and Dermo ® liner using motion analysis system. Clin Biomech.

[11] Highsmith MJ, Kahle JT, Miro RM, Orendurff MS, Lewandowski AL, Orriola JJ, Sutton B, Ertl JP (2016). Prosthetic interventions for people with transtibial amputation: systematic review and meta-analysis of high-quality prospective literature and systematic reviews. J Rehabil Res Dev.

[12] Johannesson A, Larsson GU, Oberg T (2004). From major amputation to prosthetic outcome: a prospective study of 190 patients in a defined population. Prosthet Orthot Int.

[13] Klute GK, Glaister BC, Berge JS (2010). Prosthetic liners for lower limb amputees: a review of the literature. Prosthet Orthot Int.

[14] Kristinsson O (1993). The ICEROSS concept: a discussion of a philosophy. Prosthet Orthot Int.

[15] Suyi Yang E, Aslani N, McGarry A (2019). Influences and trends of various shape-capture methods on outcomes in trans-tibial prosthetics: a systematic review. Prosthet Orthot Int.

[16] Trieb K, Lang T, Stulnig T, Kickinger W (1999). Silicone soft socket system: its effect on the rehabilitation of geriatric patients with transfemoral amputations. Arch Phys Med Rehabil.

[17] Williams RJ, Washington ED, Miodownik M, Holloway C (2018). The effect of liner design and materials selection on prosthesis interface heat dissipation. Prosthet Orthot Int.

[18] Ziegler-Graham K, MacKenzie EJ, Ephraim PL, Travison TG, Brookmeyer R (2008). Estimating the prevalence of limb loss in the United States: 2005 to 2050. Arch Phys Med Rehabil.

